# The Anxiolytic Effects of Valtrate in Rats Involves Changes of Corticosterone Levels

**DOI:** 10.1155/2014/325948

**Published:** 2014-03-20

**Authors:** Shu-Ning Shi, Jin-Li Shi, Yong Liu, Yan-Li Wang, Chun-Guo Wang, Wen-Hui Hou, Jian-You Guo

**Affiliations:** ^1^School of Chinese Materia Medica, Beijing University of Chinese Medicine, 6A Wangjing Central South Road, Chaoyang District, Beijing 100102, China; ^2^Key Laboratory of Mental Health, Institute of Psychology, Chinese Academy of Sciences, 4A Datun Road, Chaoyang District, Beijing 100101, China

## Abstract

Valtrate is a principle compound isolated from *Valeriana jatamansi* Jones, which is a Traditional Chinese Medicine used to treat various mood disorders. The aim of the present study was to investigate the anxiolytic effects of valtrate in rats. The animals were orally administered valtrate (5, 10, and 20 g/kg daily) for 10 days and exposed to open field test (OFT) and elevated plus-maze (EPM). Then the corticosterone levels in the rat serum were measured by enzyme-linked immunosorbent assay (ELISA). The valtrate (10 mg/kg, p.o.) exhibited the anxiolytic effect in rats by increasing the time and entry percentage into the open arms in the EPM and the number of central entries in the OFT. Valtrate (10 mg/kg, p.o.) significantly reduced the corticosterone level in the rat serum. Taken together, these results suggest that the valtrate has anxiolytic activity in behavioral models that might be mediated via the function of hypothalamus-pituitary-adrenal axis.

## 1. Introduction

Anxiety disorder is a common mental illness on society. Millions of people suffer from a mental or behavioral disorder [1]. Previous studies suggest that benzodiazepines are useful first-line agents for most of the anxiety disorders in the world [2]. However, they may produce fearful side effects; for example, long-term use of benzodiazepine can cause cognitive decline in the elderly [3]. In addition, a lot of patients with anxiety disorders fail to adequately respond to existing pharmacologic treatments [4]. Thus, better antianxiety drugs with greater efficacy and fewer side-effects are needed.

Traditional Chinese prescription has been commonly recognized as a safe and effective prescription in the treatment of various mood disorders in China [5].* Valeriana jatamansi* Jones was a famous Traditional Chinese Medicine used to treat anxiety disorders in clinical prescription for many years [6]. Recent study has reported that* Valeriana jatamansi* Jones exerts an anxiolytic effect by improving the frequency and time percentage of the open arm in the elevated plus maze [7]. Chemical researches have shown that it includes essential oils, iridoids, and flavonoids compounds [8], but the anxiolytic active components of* Valeriana jatamansi* Jones have not been adequately elucidated. Valtrate is a major component of* Valeriana jatamansi* Jones and has been shown to have antifungal, antitumor, and cytotoxic activities in early studies [9–12]. Currently, valtrate at a high dose has been found to have sedative properties by inhibiting spontaneous motion and increasing the sleeping number induced by pentobarbital sodium in mice [13]. Therefore, these results raises the possibility of the anxiolytic effect of valtrate as the primary antianxiety components in* Valeriana jatamansi* Jones. However, the anxiolytic effect of valtrate and the mechanism have not been reported.

Therefore, in the present study, we investigated the anxiolytic potential of valtrate isolated from* Valeriana jatamansi* Jones in rats. The paradigms we selected here to detect the anxiolytic effect of valtrate are two famous tests of anxiety: the open field test (OFT) and the elevated plus maze test (EPM), which have shown good sensitivity to anxiolytic drugs. The EPM is a well-established animal model for testing anxiolytic drugs [14] because of its natural stimulus, such as a fear of a new, bright, and open space and the fear of balancing on a relatively narrow raised surface [15]. The OFT has gained popularity as a model of anxiety, which is based on the rodents' natural tendency to stay near the perimeters of a novel environment [16] and the aversion of rodents for open and illuminated spaces [17]. The animals were tested in the OFT and EPM. After the behavior test, we determined whether valtrate altered the serum corticosterone response to stress induced by exposure to the two models.

## 2. Material and Methods

### 2.1. Animals

60 male 8-week-old Sprague-Dawley rats (150–170 g) were obtained from the Laboratory Animal Center of the Academy of Military Medical Sciences and used for this study. Each animal was housed in individual cages under controlled temperature (22 ± 1°C) and a 12 h/12 h light/dark cycle (lights on at 07:00 AM–19:00 PM) with free access to food and water. The experimenter handled the animals daily to acclimate them to the manipulation. The experimental procedures were approved by the Institutional Animal Care and Use Committee of the Institute of Psychology of the Chinese Academy of Sciences and in accordance with the National Institutes of Health Guide for Care and Use of Laboratory Animals.

### 2.2. Plant Material and Isolation of Valtrate

*Valeriana jatamansi* Rhizoma et Radix was purchased from a commercial source in Yunnan province, China. The identity of the herbal medicine was confirmed by Professor Shi Jin-li, a researcher in the Department of Pharmacognosy, Beijing University of Chinese Medicine. Voucher specimens were deposited at the Herbarium of School of Chinese Materia Medica, Beijing University of Chinese Medicine.

**Figure 1:**
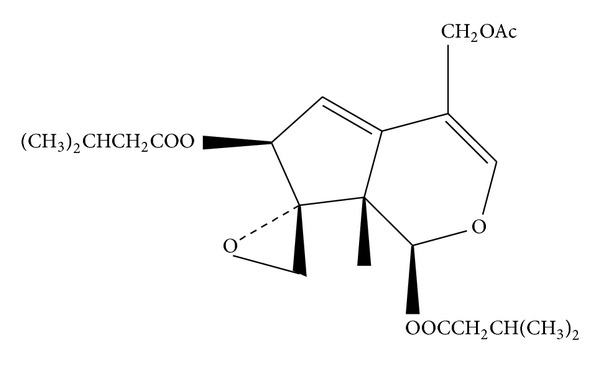
Structure of valtrate.

Jatamana Valeriana Rhizome was homogenized to coarse powder (8 kg) and soaked in aqueous ethanol (95%, 12 L, v/v) three times at room temperature, and the combined alcoholic extract was filtered and evaporated under reduced pressure to yield a residue. The concentrated extract was then subjected to chromatographic separation on AB-8 macroporous adsorption resin with 70%, 80%, and 90% EtOH-H_2_O to give three fractions. Three fractions were subjected to chromatography on silica gel eluted with petroleum ether-ethyl acetate (20 : 1, 10 : 1, 8 : 1), then The fractions were combined based on the TLC analysis. We got ten compounds; the valtrate (Figure 1) was an oily matter identified by spectroscopic methods (UV, IR, ESI-MS, ^1^H NMR, and ^13^C NMR). The purity of valtrate was determined by HPLC analysis, which was identified by comparing with a standard specimen (National Institute for the Control of Pharmaceutical and Biological Products, Beijing, China). The sample was chromatographed under the following chromatographic conditions: Chromatographic column: Agilent Extend C18 column, 5 *μ*m, 250 × 4.6 mm; Mobile phase: gradient elution by acetonitrile-distilled water (68%–32%); Flow rate: 1 mL/min; Column temperature: 30°C with UV detection at 254 nm.

### 2.3. Drugs and Treatment

Diazepam was obtained from Yimin Pharmaceutical Factory of Beijing. All drugs were prepared immediately before use and were given orally in a volume of 1 mL/100 g body weight for 10 days. diazepam at the dose of 1 mg/kg [18] was chosen as a positive control drug. Diazepam and valtrate were both dissolved in 0.5% Tween-80 solution. For vehicle group, distilled water which contained 0.5% Tween-80 was administered at the same volume. In this study, the rats were administered valtrate or diazepam 60 and 30 min before the test, respectively. The Elisa kit was obtained from R&D. All experiments were carried out in quiet room under dim red light between 8:00 a.m. and 14:00 p.m. on the 10th day of treatment.

### 2.4. Open Field Test

The OFT apparatus was a 180 cm diameter cylinder with 60 cm high walls. The center of the bottom of the apparatus had a 52 cm diameter section. As previously described [19], all rats were acclimatized to the test room for 1 h. The rats were placed into the field at the same point against the wall and allowed to freely explore the apparatus for 10 min. The total path length, the number of central entries, and the time spent in the center were recorded by an automatic video tracking system. OFT was performed 60 min after the final treatment of valtrate and 30 min after the diazepam. After each trial, the apparatus was wiped clean with a 10% ethanol solution.

### 2.5. Elevated Plus Maze

Immediately after the OFT, anxiolytic activity was measured using the EPM, which was consisted of two open arms (50.8 cm × 10.2 cm × 1.3 cm) and two closed arms (50.8 cm × 10.2 cm × 40.6 cm) that extended from a central platform (10.2 cm × 10.2 cm). The maze was elevated to a height of 72.4 cm above the floor. The entire maze was constructed of clear Plexiglas [20]. Each rat was placed on the central square facing an open arm and allowed to freely explore the maze for 5 min. Arm entries were defined as the entry of all four paws into an arm. A computer recorded the time spent on and number of entries into the open and closed arms by means of infrared photocells. The apparatus was wiped clean with a 30% ethanol solution and dried after each subject.

### 2.6. Determination of Serum Corticosterone

**Figure 2:**
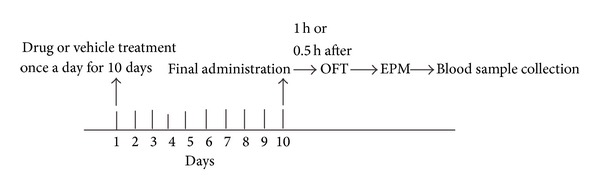
Experimental schedule. Experimental schedule, described in Material and Methods section, involved the OFT, EPM test, and the collection of blood sample.

10 min after the completion of the two behavioral tests, the rats were sacrificed by decapitation; then trunk blood was collected among the five groups to avoid any substantial time lag in samples collection. Samples were centrifuged at 3000 r·min^−1^ for 15 min at 4°C and supernatants were stored at −20°C until analysis. The content of corticosterone was determined by a commercially available enzyme-linked immunosorbent assay (ELISA) kit according to the manufacturer's instructions. The absorbance of each sample was measured at a wavelength of 450 nm and the results are presented as ng/mL. All procedures of the experiment were shown in Figure 2.

### 2.7. Statistical Analysis

The data were expressed as mean ± SEM. The statistical analysis was carried out by one-way analysis of variance (ANOVA) following Student-Newman-Keul's post-hoc test using Prism 5.0 (Graphpad Software, Inc). Probability values lower than 0.05 were considered statistically significant.

## 3. Results

### 3.1. Assaying of Valtrate by HPLC

**Figure 3:**
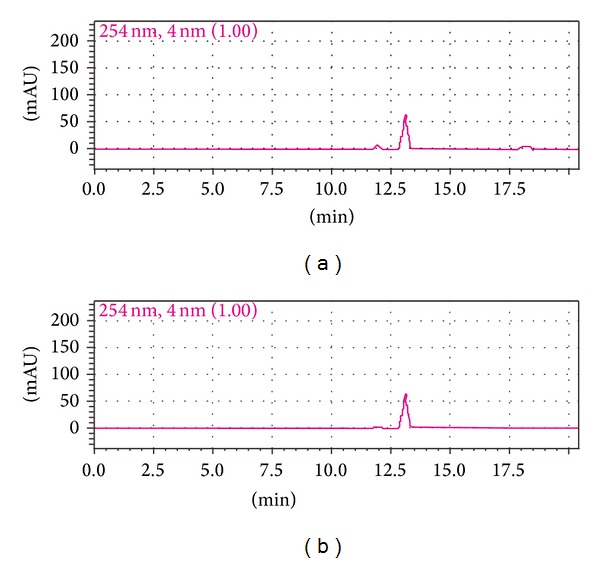
HPLC profile of valtrate using acetonitrile-distilled water (68%–32%) at 1 mL/min on a Agilent Extend C18 column, 5 *μ*m, 250 × 4.6 mm, 30°C with UV detection at 254 nm. (a) The sample of valtrate. (b) The standard specimen of valtrate.

The results suggest that the purity of product can reach 99% (see Figure 3).

### 3.2. Effects of Valtrate on Open Field Test in Rats

**Figure 4:**
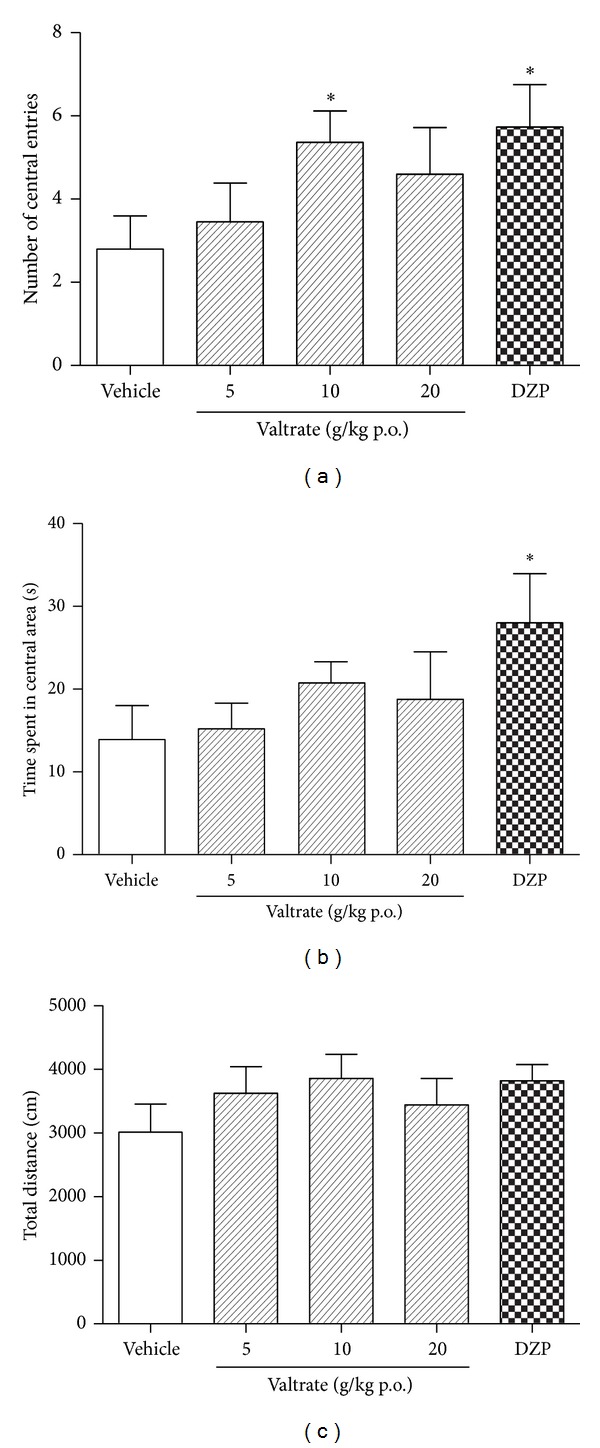
Effect of valtrate on the behavior of rat in the OFT in a 10 min session in the open field performed 1 h after the administration of vehicle (p.o.), valtrate (5, 10, and 20 mg/kg, p.o.), and 0.5 h after the administration of diazepam (1 mg/kg, p.o.). (a) Number of central entries, (b) time spent in central area, and (c) total distance. Columns represent the means ± SEM, *n* = 12 rats. *P < 0.05 compared to the vehicle group.

**Figure 5:**
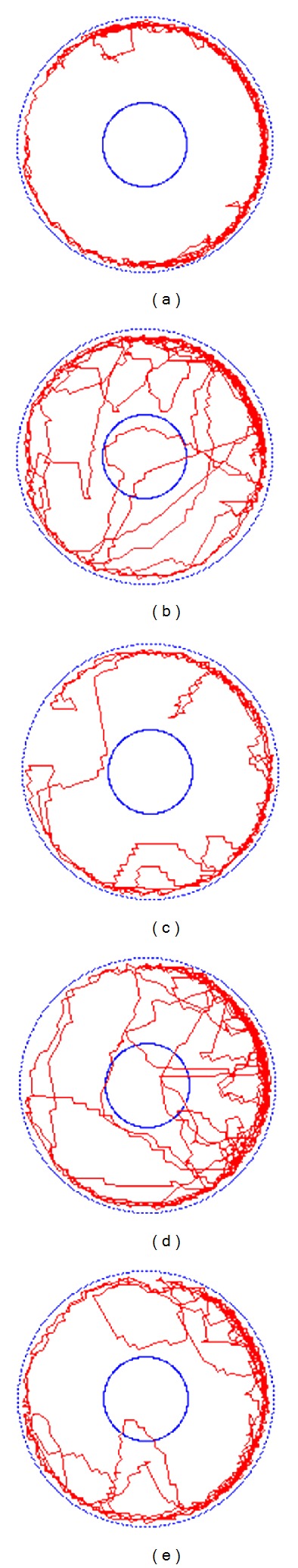
Locus diagram of OFT. (a) Vehicle, (b) DZP, and (c) Valtrate 5 mg/kg, (d) valtrate 10 mg/kg, and (e) valtrate 20 mg/kg.

The results for the OFT are shown in Figure 4. Analyses demonstrated significant effects on number of center entries (*F* (4, 55) = 3.541, *P* < 0.05) and time spent in central area (*F* (4, 55) = 3.127, *P* < 0.05); further analyses showed that valtrate at dose of 10 mg/kg significantly increased the entries in central area (*P* < 0.05). Valtrate at the dose of 20 mg/kg did not significantly increase the entries in central area (*P* > 0.05). Diazepam significantly increased the number of center entries (*P* < 0.05) and the time spent in central area (*P* < 0.05). All of the doses of valtrate did not significantly increase the time spent in central area (*P* > 0.05). No difference in total path length was observed among the five groups (*F* (4, 55) = 1.207, *P* > 0.05). Locus diagram of open field test of every group is shown in Figure 5.

### 3.3. Effects of Valtrate on Elevated Plus Maze in Rats

**Figure 6:**
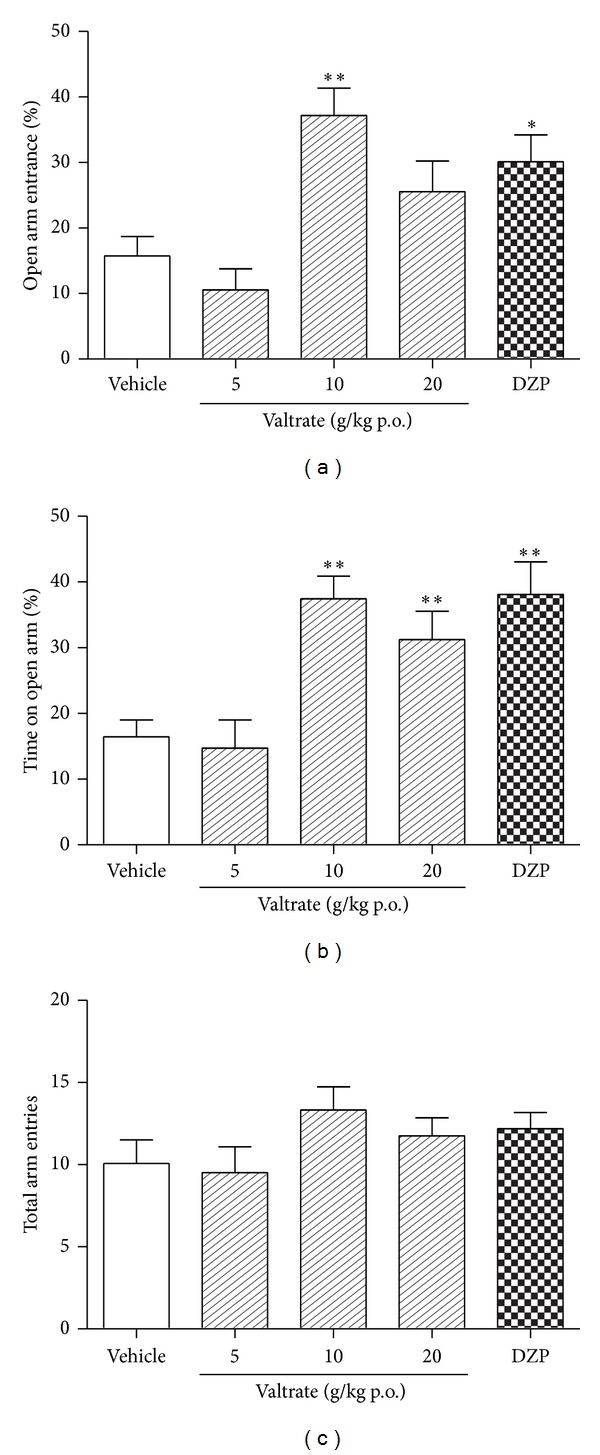
Behavioural performance of rat registered in a 5 min session in the EPM performed 1 h after the administration of vehicle (p.o.), valtrate (5, 10, and 20 mg/kg, p.o.), and 0.5 h after the administration of diazepam (1 mg/kg, p.o.). (a) Percentage of number of entries into the open arm, (b) percentage of time spent into the open arms, and (c) total arm entries. Columns represent the means ± SEM, *n* = 12 rats. *P < 0.05, **P < 0.01 compared to the vehicle group.

As shown in Table 1 and Figure 6, the ANOVA indicated significant effects on percentage of time spent on the open arm (*F* (4, 55) = 7.755, *P* < 0.01) and open arm entries (*F* (4, 55) = 6.054, *P* < 0.01). Compared to vehicle group, valtrate at the dose of 10 mg/kg significantly increased the percentage of time spent in the open arms and entry percentage into the open arms in the elevated plus maze (*P* < 0.01; *P* < 0.01), and valtrate at the dose of 20 mg/kg increased the percentage of time spent in the open arms of the maze (*P* < 0.01) but did not increase percentage of open arm entries (*P* > 0.05). Diazepam also significantly increased the percentage of time spent on open arms (*P* < 0.01) and percentage into the open arms (*P* < 0.05). No difference was observed in total arm entries among groups (*F* (4,55) = 1.042, *P* > 0.05).

### 3.4. The Level of Serum Corticosterone

**Figure 7:**
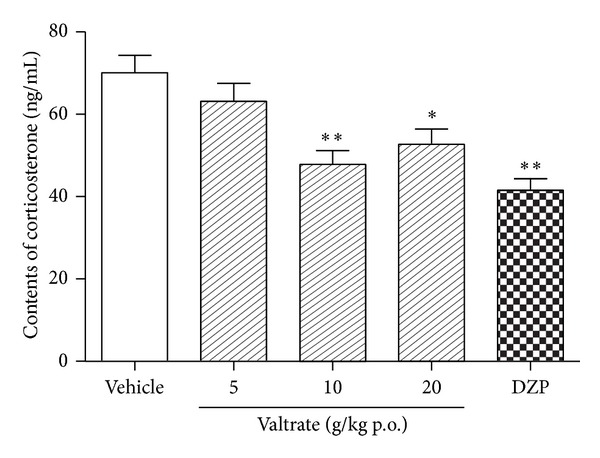
Effects of bergamot valtrate (5, 10, and 20 mg/kg, p.o.) and diazepam (1 mg/kg, p.o.) compared with vehicle groups on serum corticosterone after the behavior test. Columns represent the means ± SEM, *n* = 12. *P < 0.05, **P < 0.01 compared to the vehicle group.

As seen in Figure 7, the data show that administration of valtrate at the dose of 10 mg/kg and 20 mg/kg dose reduced the corticosterone level (*P* < 0.01, *P* < 0.05). Similarly, serum corticosterone levels of rats treated with diazepam were lower than those of the vehicle group (*P* < 0.01).

## 4. Discussion

The present study was performed to analyze the behavioral effects of anxiolytic valtrate isolated from* Valeriana jatamansi* Jones, using two behavioural measurements of anxiety, OFT, and EPM. The results showed that valtrate exhibited anxiolytic-like activity and did not induce sedative side effects. We also found that valtrate could attenuate HPA axis activity by reducing the corticosterone level.

Valtrate was successfully isolated from subterranean parts of subterranean parts of various Valeriana species for the first time by Thies [21]. Our laboratory developed a high efficiency and practicality method for purifying Valtrate from* Valeriana jatamansi* Jones with AB-8 macroporous adsorption resin. The resin yielded the best efficiency when the concentration of the extraction was 3.5 mg/mL, the 70% ethanol acted as the eluant, and the eluting speed was two column volumes per hour. AB-8 macroporous adsorption resin significantly increased the purity of valtrate (99%), with advantage of high absorption, high elution rate, and low expense.

Hall originally described the OFT for the study of emotionality in rats [22], which is one of the most popular procedures in animal psychology and has been widely used to assess anxiety, emotionality, or responses to stress in animals [23, 24]; the test is based on the rodents' natural tendency to stay near the perimeters of a novel environment and the aversion of rodents for open and illuminated spaces. The number of central entries or the time spent in the center area served as indices of anxiety and the distance was considered an index of locomotor activity [25, 26]. Rats treated with valtrate at the dose of 10 mg/kg significantly increased the number of center entries and the total distance was not significantly affected. Therefore, valtrate (10 mg/kg) has a significant anxiolytic-like effect in this paradigm.

To further strength these data, we tested the anxiolytic-like effects of these treatments in EPM, Which is a classical animal analog for anxiolytic drugs and can play a key role in the screening of anxiolytic drugs on the central nervous system currently [27, 28]. Normally, rodents tend to avoid open areas of the maze and a preference for sections enclosed by protective walls. Anxiolytic drugs shift the behavioral response toward exploration of the open arms [29]. The percentage entries into the open arms and time spent in the open arms are generally used as indices of anxiety and drugs increasing these measures show anxiolytic properties. The number of entries into the total arms was considered an index of locomotor activity. In the present study, the rats were treated with the higher doses of valtrate (10 mg/kg and 20 mg/kg) for 10 days, and anxiety-like behavior in the EPM was significantly attenuated, without altering the number of total arm entries, suggesting that valtrate induces specific anxiolytic-like effects.

The alcohol extract from* Valeriana jatamansi* Jones (0.3, 0.6, 0.9 g/kg) could increase the open entries percent and open time percent in the EPM [30]. As the content of valtrate in alcohol extract from* Valeriana jatamansi* Jones is about 2.87%, 5 mg/mL valtrate in rat is equivalent to 0.3 g/mL alcohol extract of* Valeriana jatamansi* Jones in mice in terms of equal valtrate efficacy. Thus, the doses of valtrate (5, 10, and 20 mg/kg) were chosen in this study. We also performed an acute experiment to study the anxiolytic effect of valtrate, and valtrate did not affect the behavior tests in EPM and OFT (data not shown). Therefore, the valtrate was administered with ten days. Diazepam is a classical drug to treat anxiety and was chosen as a positive drug in this study. As expected, diazepam had a significant anxiolytic-like effect in both EPM and OFT. The anxiolytic effect of the valtrate (10 mg/kg) was almost equivalent to that of diazepam (1 mg/kg) in EPM and the number of central entries in OFT. Although valtrate did not increase the time spent on the center of the open field compared to the vehicle group, there were marginal significant differences between the valtrate and the vehicle group on these tests. In addition, both valtrate (10 mg/kg) and diazepam (1 mg/kg) could reduce the corticosterone levels after behavior tests. Therefore, we thought that the anxiolytic effect of valtrate (10 mg/kg) was almost equivalent to diazepam (1 mg/kg).

The drugs (valtrate or diazepam) in this study were administered 60 and 30 min before the test; there should be a control group for each drug treatment. We compared these two vehicle groups (administrated 30 min and 60 min, resp.). The rats of these groups were exposed to the same procedure. The results suggested that there was no difference between the two groups (data not shown). To avoid too much groups in the present study, one vehicle group (60 min before test) was used as control.

It should be noted that the intermediate dose of valtrate was the most effective in decreasing anxiety-like behavior tests and corticosterone concentrations. One possible reason may be that the highest dose of valtrate or its metabolites may act as an inducer for hepatic microsomal enzyme, which can increase metabolism of the drug and result in reducing curative effect. In addition, valtrate (10 mg/kg and 20 mg/kg) was able to decrease corticosterone concentration (*P* < 0.01 and *P* < 0.05, resp.), but this effect was not observed in the behavior tests. Although valtrate at the dose of 20 mg/kg did not increase percentage of open arm entries (*P* = 0.052) and the number of central entries (*P* = 0.058), there were marginal significant differences between the valtrate (20 mg/kg) and the vehicle group on these two tests. Moreover, individual differences among rats in the highest dose valtrate (20 mg/kg) group are larger than these in intermediate dose valtrate (10 mg/kg) group. The same situation was observed in the open field test. However, valtrate at the dose of 10 mg/kg and 20 mg/kg did not increase the time spent on the center of the open field compared to the vehicle group (*P* = 0.057 and *P* = 0.063, resp.). There were marginal significant differences between the valtrate group (10 mg/kg or 20 mg/kg) and the vehicle group in the time spent in the center area.

Neuroendocrine system plays a key role in the stability of the body environment. Hormones change these neurons' network by making change in information and altering the neurotransmitter between cells in cell level, thus affecting the central nervous system function [31]. It is well known that hypothalamic-pituitary-adrenocortical (HPA) axis activation is a key component of the physiological response to stress and anxiety. The HPA axis is activated by stress; then corticosterone is released from the adrenal gland. The stress hormone corticosterone was measured to investigate the response of the HPA axis to valtrate. Research suggests that the exposure of rodents to the standard elevated plus maze activates the HPA axis, leading to an enhancement of plasma corticosterone [32]. In addition, there was a peak in corticosterone secretion which occurs 5 to 10 min after exposure to two different anxiety/fear tests [33]. It has been reported that* Valeriana jatamansi* Jones extract played a role in antianxiety via regulation of the HPA axis [30]. In the present study, as the stress hormone corticosterone was measured as the rats were subjected to both EPM and OF tests. These two tests were performed in two adjacent rooms, the delay time was less than one minute, and we thought that these procedures might not influence the effectiveness of the drug or plasma corticosterone concentrations, which were also used by other researchers [34, 35]. Our data showed that valtrate (10 mg/kg), a dose which produced anxiolytic activity in the behavioural experiments, attenuated the activity of HPA axis by reducing the corticosterone response to the stress of exposure to the elevated plus maze. These findings indicate that the decreased anxiety-related behaviours may be related to the attenuation of HPA axis activity.

You et al. had reported the anxiolytic-like effects of compound* Valeriana jatamansi* Jones in mice [36]. Compound* Valeriana jatamansi* Jones is composed of Valerianae Jatamansi Rhizoma et Radix, Ziziphi Spinosae Semen, and Albiziae Cortex and Junci Medulla (in a ratio of 12 : 9 : 9 : 1). They reported that the compound* Valerianae Jatamansi* Jone has anxiolytic effects but no sedative effect at dose of 2.4 and 4.8 g/kg. As the content of valtrate in* Valeriana jatamansi* Jones is about 1.8%, 2.4 g and 4.8 g valtrate compound might contain valtrate 16.74 mg and 33.48 mg, respectively. In our study, valtrate at the dose of 10 mg/kg and 20 mg/kg has anxiolytic-like effect in rats. Thus, valtrate might be the main component to possess the anxiolytic-like effect of compound* Valeriana jatamansi* Jones. However, it is very interesting to contrast some pharmacological property of valtrate and* Valeriana jatamansi* Jones (or compound* Valeriana jatamansi* Jones) on behavior and plasma corticosterone. In addition, we did not measure the corticosterone levels in treated nonstressed rats. As the present procedure is characterized by two factors (stress and treatment), the corticosterone levels in treated nonstressed rats could strongly improve our present results and will be added in the future study.

The EPM test is considered one of the most widely validated tests for assaying new benzodiazepine-like anxiolytic agents [37]. GABA is the most important inhibitory neurotransmitter in the human central nervous system. Most of GABA receptors have separate modulatory sites sensitive to benzodiazepines. It is well known that the GABA mediated inhibition of the HPA axis at the level of the paraventricular nucleus of the hypothalamus [38]. As a consequence, we hypothesized that the decreased corticosterone levels by valtrate may also be related to the GABAergic neurotransmission.

## 5. Conclusions

In conclusion, the present study indicates that valtrate exhibits anxiolytic-like profiles in the elevated plus maze test and the open field test. Valtrate also attenuated HPA axis activity by reducing the corticosterone level.

## Figures and Tables

**Table 1:** Effect of diazepam and valtrate on the behavior of rats in the elevated plus-maze test.

Group	Dose (mg/kg)	Open arm entries	Closed arm entries	Total arm entries	Time in open arms (s)	Time in closed arms (s)
Vehicle	—	3.07 ± 0.59	9.16 ± 0.95	10.07 ± 1.45	48.41 ± 4.36	189.94 ± 10.57
Diazepam	1	4.80 ± 0.73	9.70 ± 0.76	12.20 ± 0.97	98.92 ± 10.07	138.55 ± 9.54
Valtrate	5	2.00 ± 0.29	10.71 ± 0.97	9.50 ± 1.59	52.21 ± 8.09	183.31 ± 15.45
	10	6.36 ± 1.16	10.55 ± 1.23	13.33 ± 1.41	87.20 ± 10.20	143.13 ± 8.92
	20	4.22 ± 0.47	8.78 ± 0.70	11.75 ± 1.11	82.01 ± 8.69	147.51 ± 8.68

## References

[1] Reynolds EH (2003). Brain and mind: a challenge for WHO. *The Lancet*.

[2] Woods JH, Katz JL, Winger G (1992). Benzodiazepines: use, abuse, and consequences. *Pharmacological Reviews*.

[3] Paterniti S, Dufouil C, Alpérovitch A (2002). Long-term benzodiazepine use and cognitive decline in the elderly: the epidemiology of vascular aging study. *Journal of Clinical Psychopharmacology*.

[4] Ravindran LN, Stein MB (2010). The pharmacologic treatment of anxiety disorders: a review of progress. *The Journal of Clinical Psychiatry*.

[5] Guo J-Y, Han C-C, Liu Y-M (2010). A contemporary treatment approach to both diabetes and depression by cordyceps sinensis, Rich in Vanadium. *Evidence-Based Complementary and Alternative Medicine*.

[6] Zheng HZ, Shi JL (2012). Anxiolytic-like effects of compound zhi zhu xiang Capsule about anxiety disorders of learning in clinical. *Modern Journal of Integrated Traditional Chinese and Western Medicine*.

[7] Yan ZY, Zhang TE, Peng J, Zhang ZP, Qin JZ, Chen C (2008). Effect of the extract of Valeriana jatamansi Jones on the ethology and neurotransm itter in the brain in the anxietymodel of rat. *Pharmacology and Clinics of Chinese Materia Medica*.

[8] Li S-H, Yan Z-Y (2012). Research of iridoids from Valeriana jatamansi Jones. *Chinese Journal of New Drugs*.

[9] Fuzzati N, Wolfender JL, Hostettmann K, Msonthi JD, Mavi S, Molleyres LP (1996). Isolation of antifungal valepotriates from Valeriana capense and the search for valepotriates in crude Valerianaceae extracts. *Phytochemical Analysis*.

[10] Keochanthala-Bounthanh C, Beck JP, Haag-Berrurier M, Anton R (1993). Effects of two monoterpene esters, valtrate and didrovaltrate, isolated from *Valeriana wallichii*, on the ultrastructure of hepatoma cells in culture. *Phytotherapy Research*.

[11] Keochanthala-Bounthanh C, Haag-Berrurier M, Beck JP, Ant on R (1990). Effects of thiol compounds versus the cytotoxicity of valepotriates on cultured hepatoma cells. *Planta Medica*.

[12] Bounthanh C, Bergmann C, Beck JP, Haag-Berrurier M, Anton R (1981). Valepotriates, a new class of cytotoxic and antitumor agents. *Planta Medica*.

[13] Chen L, Kang LP, Qin LP, Zheng HC, Guo C (2003). The sedative activity of the Valepotriates in mice. *Chinese Traditional Patent Medicine*.

[14] Dawson GR, Tricklebank MD (1995). Use of the elevated plus maze in the search for novel anxiolytic agents. *Trends in Pharmacological Sciences*.

[15] Millan MJ (2003). The neurobiology and control of anxious states. *Progress in Neurobiology*.

[16] Brotto LA, Barr AM, Gorzalka BB (2000). Sex differences in forced-swim and open-field test behaviours after chronic administration of melatonin. *European Journal of Pharmacology*.

[17] Belzung C, Griebel G (2001). Measuring normal and pathological anxiety-like behaviour in mice: a review. *Behavioural Brain Research*.

[18] Wang YL, Shi JL, Yong L, Zhao R, Zhai YJ, Guo JY (2012). Anxiolytic-like effects of compound Zhi Zhu Xiang in rats. *Evidence-Based Complementary and Alternative Medicine*.

[19] Polissidis A, Chouliara O, Galanopoulos A (2010). Individual differences in the effects of cannabinoids on motor activity, dopaminergic activity and DARPP-32 phosphorylation in distinct regions of the brain. *The International Journal of Neuropsychopharmacology*.

[20] Lister RG (1990). Ethologically-based animal models of anxiety disorders. *Pharmacology and Therapeutics*.

[21] Thies PW (1968). Die konstitution der valepotriate. Mitteilung über die wirkstoffe des baldrians. *Tetrahedron*.

[22] Walsh RN, Cummins RA (1976). The open-field test: a critical review. *Psychological Bulletin*.

[23] Sherif F, Harro J, El-Hwuegi A, Oreland L (1994). Anxiolytic-like effect of the GABA-transaminase inhibitor vigabatrin (gamma-vinyl GABA) on rat exploratory activity. *Pharmacology Biochemistry and Behavior*.

[24] Ang HH, Cheang HS (1999). Studies on the anxiolytic activity of Eurycoma longifolia Jack roots in mice. *Japanese Journal of Pharmacology*.

[25] Prut L, Belzung C (2003). The open field as a paradigm to measure the effects of drugs on anxiety-like behaviors: a review. *European Journal of Pharmacology*.

[26] Rentesi G, Antoniou K, Marselos M, Fotopoulos A, Alboycharali J, Konstandi M (2010). Long-term consequences of early maternal deprivation in serotonergic activity and HPA function in adult rat. *Neuroscience Letters*.

[27] Bhattacharya SK, Mitra SK (1991). Anxiolytic activity of Panax ginseng roots: an experimental study. *Journal of Ethnopharmacology*.

[28] Hasenöhrl RU, Nichau C, Frisch C (1996). Anxiolytic-like effect of combined extracts of Zingiber officinale and ginkgo biloba in the elevated plus-maze. *Pharmacology Biochemistry and Behavior*.

[29] Guo J-Y, Li C-Y, Ruan Y-P (2009). Chronic treatment with celecoxib reverses chronic unpredictable stress-induced depressive-like behavior via reducing cyclooxygenase-2 expression in rat brain. *European Journal of Pharmacology*.

[30] Yan ZY, Zhang TE, Xiao T (2010). Anti-anxiety effect of Valeriana jatamansi Jones extract via regulation of hypothalamus-pituitary-adrenal axies. *Neural Regeneration Research*.

[31] Basedovsky H, Sorkin E (1977). Network of immune neuroendocrine interactions. *Clinical & Experimental Immunology*.

[32] Rodgers RJ, Haller J, Holmes A, Halasz J, Walton TJ, Brain PF (1999). Corticosterone response to the plus-maze: high correlation with risk assessment in rats and mice. *Physiology and Behavior*.

[33] Amaral VCS, Santos Gomes K, Nunes-de-Souza RL (2010). Increased corticosterone levels in mice subjected to the rat exposure test. *Hormones and Behavior*.

[34] Venâncio ET, Rocha NFM, Rios ERV (2011). Anxiolytic-like effects of standardized extract of Justicia pectoralis (SEJP) in mice: involvement of GABA/benzodiazepine in receptor. *Phytotherapy Research*.

[35] Miguel CJ, Juan FRL, Maria LRP (2012). The aqueous crude extract of Montanoa frutescens produces anxiolytic-like effects similarly to diazepam in Wistar rats: involvement of GABAA receptor. *Journal of Ethnopharmacology*.

[36] You JS, Peng M, Shi JL (2012). Evaluation of anxiolytic activity of compound Valeriana jatamansi Jones in mice. *BMC Complementary and Alternative Medicine*.

[37] Pellow S, Chopin P, File SE, Briley M (1985). Validation of open:closed arm entries in an elevated plus-maze as a measure of anxiety in the rat. *Journal of Neuroscience Methods*.

[38] Cullinan WE, Ziegler DR, Herman JP (2008). Functional role of local GABAergic influences on the HPA axis. *Brain Structure and Function*.

